# Aquagenic urticaria in twins

**DOI:** 10.1186/1939-4551-6-2

**Published:** 2013-01-31

**Authors:** Anneke C Kai, Carsten Flohr

**Affiliations:** 1 Cutaneous Allergy Department, St John’s Institute of Dermatology, Guy’s & St Thomas’ NHS Foundation Trust, Westminster Bridge Road, SE1 7EH, London, UK; 2 King’s College London, Strand, WC2R 2LS, London, UK

**Keywords:** Urticaria, Aquagenic

## Abstract

We describe the case of 18 year old twin brothers who presented to our unit with a 3 year history of aquagenic urticaria. This rare form of urticaria usually presents within an hour of contact with water. The aetiology is unknown. Most cases are sporadic but there are a small number of familial cases in the medical literature. A specific genetic mutation has not yet been found. To our knowledge, this is the first report of aquagenic urticaria in monozygotic twins, further supporting a genetic component to this disease.

## Background

We describe the case of 18 year old twin brothers who presented with aquagenic urticaria. This is a rare form of urticaria which usually presents within an hour of contact with water. The aetiology is not understood. Most cases are sporadic but there are a small number of familial cases in the medical literature although no specific genetic mutation has been found yet. To our knowledge, this is the first report of aquagenic urticaria in monozygotic twins, further supporting a genetic component to this disease.

## Case presentation

**Figure 1:**
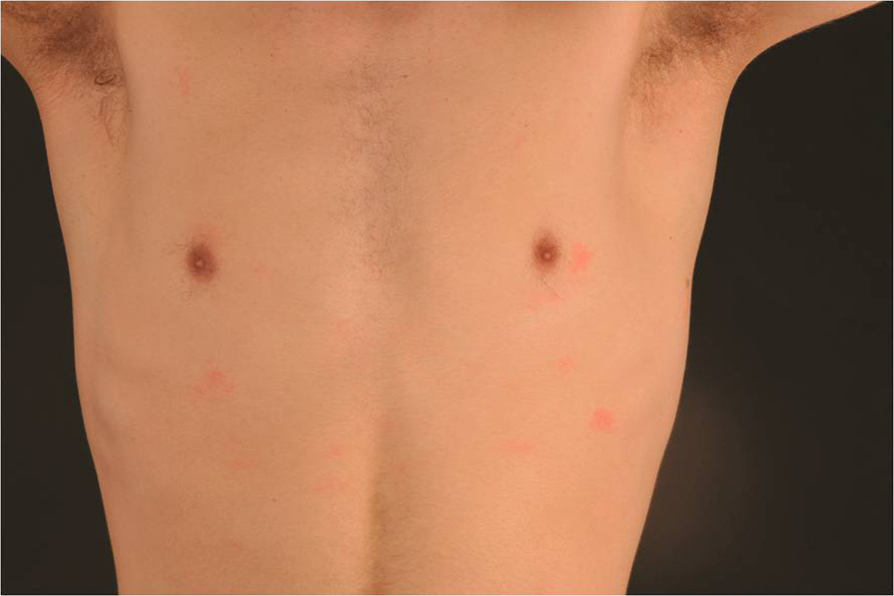
Wheals on torso after water exposure.

**Figure 2:**
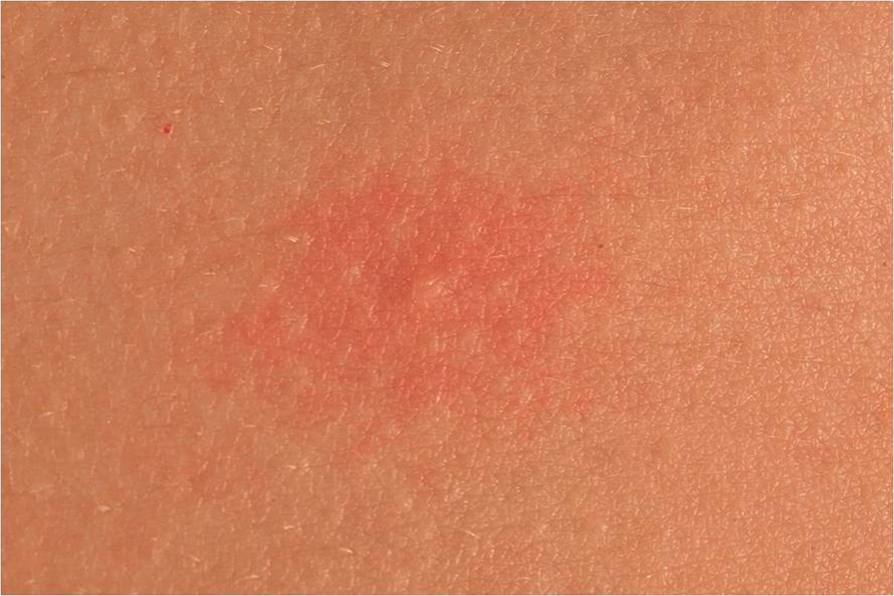
Close-up view of wheal on torso after water exposure.

18 year old identical twin brothers were referred to our tertiary referral centre urticaria clinic with a three year history of aquagenic urticaria. They were both keen triathletes and were therefore frequently exposed to water. Swimming or showering would induce a prodrome of skin pruritus followed by the development of wheals over the trunk approximately 15 minutes after the initial contact with water. The wheals were induced by both hot and cold water. They would resolve spontaneously after about one hour, leaving no residual mark. Neither brother had ever experienced any associated angioedema. Exercise such as running would only induce itching. There was no history of atopy and both brothers were in good general heath and were not taking any regular medications. The family history was unknown as the brothers had been adopted. On examination, the skin of both brothers was normal but we were able to induce the classical wheals following exposure to water in a shower in our clinic (Figures 1 and 2). The symptoms were controlled by taking prophylactic cetirizine 10mg once per day.

## Discussion

Aquagenic urticaria is a rare form of urticaria which affects women more commonly than men. Symptoms often start in puberty. Wheals usually develop within 30 to 60 minutes of contact with water, irrespective of the temperature. Differential diagnoses include aquagenic pruritus, where exposure to water induces pruritus but no visible skin lesions, and cholinergic urticaria, where wheals develop in response to heat, exertion, sweating or emotional stress. The aetiology is not understood. It has been suggested that water interacting with sebum or sebaceous glands can produce a toxin causing degranulation of mast cells, leading to histamine release and wheal formation. An alternative explanation has been that water-soluble antigens in the epidermis diffuse into the dermis and cause a histamine mediated reaction. Oral antihistamines are used as first line treatment, up to four times the standard single daily dose. Successful treatment with UVB and PUVA has also been reported.

Although almost all cases of aquagenic urticaria are sporadic, there are a small number of familial cases in the medical literature [1-7]. Some of these cases have been reported in association with other conditions, a few of which are genetically linked. For example, Pitarch et al. [1] published a case of three affected female siblings all with co-existent. Bernard-Soulier Syndrome, a rare autosomal recessive disorder causing a prolonged bleeding time for which more than 30 mutations of the GPIbα, GPIbβ and GPIX genes have been described [8]. These genes map to chromosomes 17p12, 22q11.2 and 3q21 respectively. They postulated that although the association could be a coincidence, it might constitute an association of genetic loci.

Treudler er al [2] reported an association of familial aquagenic urticaria with familial lactose intolerance over three generations. They described a male patient with aquagenic urticaria whose grandmother, mother, aunt and cousin were all affected. The patient, mother and grandmother were also affected by familial lactose intolerance. Familial lactose intolerance is caused by a deficiency of the enzyme lactase which is encoded on chromosome 2, a different genetic locus to the mutations causing Bernard-Soulier syndrome (chromosomes 17, 22 and 3).

## Conclusions

To the best of our knowledge, this is the first case report of aquagenic urticaria in monozygotic twins, further supporting the notion that there is a genetic component to this type of urticaria, as suggested by others. It is hoped that new generation sequencing/exome sequencing will be able to shed further light on the genetic traits involved.

## Consent

Written informed consent was obtained from the patients for publication of this Case Report and any accompanying images. A copy of the written consent is available for review by the Editor-in Chief of this journal.

## Competing interests

The authors declare that they have no competing interests.

## Authors’ contributions

CF provided the case history. AK carried out the literature review and wrote the discussion. All authors read and approved the final manuscript.
